# Gene Therapy Protocols

**DOI:** 10.1038/sj.bjc.6604956

**Published:** 2009-03-17

**Authors:** O Danos

**Affiliations:** 1 UCL Gene Therapy Consortium, UCL Cancer Institute, University College London, 72 Huntley Street, London WC1E 6BT, UK

In his introduction to the two volumes of *Gene Therapy Protocols*, from the prestigious *Methods in Molecular Biology* series, Joseph M Le Doux reminds us that the idea behind gene therapy is deceivingly simple: *‘*to deliver genetic material to cells that will slow down or halt the progression of disease, or to help repair or degenerate damaged or lost tissues*’*.

One may add that although traditional drugs act by direct and transient modification of a biological target, the original proposal of gene therapy is to reprogramme the cells. The idea must have been around in the minds of many a postmodern Jules Verne, ever since Avery, Mc Leod and Mc Carthy showed in 1944 that the phenotype of bacteria could be modified by DNA transfer. Yet, major scientific breakthroughs, including recombinant DNA, had to take place before gene isolation, modification and transfer became a reality, an indispensable tool for modern biology and a technology with great potential for medical application.

The simple idea of gene therapy may be applied to the whole of medicine, from sport-related injuries to neurodegenerative diseases and cancer. Ever since the first clinical trial in 1989, the prospect of gene therapy has caused high expectations. Proofs of concept have accumulated rapidly in animal models of human diseases, and clinical studies have shown biological responses or clinical efficacy in human patients with genetic diseases affecting the immune system, skin or blood clotting. Positive data have also been reported in patients with AIDS, melanoma and Parkinson's disease.

The two volumes of *Gene Therapy Protocols* are dedicated to the various aspects of gene transfer technologies. Gene transfer can be obtained using physical methods, such as electric fields or ultrasounds, that create a temporary access to the intracellular milieu for exogenous nucleic acids. It can also be mediated by vectors that are able to package and deliver genetic information. Vectors can either be formed by synthetic macromolecules, such as polycations, cationic lipids or peptides, or by recombinant viral particles endowed with the sophisticated cell invasion capacities of the parent virus. Synthetic vectors may offer the advantage of simpler manufacturing processes and industrial scale-up when compared with viral vectors that involve complex biotechnological procedures and cell culture. Yet, the efficacy of viral vectors often designates them as the only tools available for clinical application.

A total of 40 short chapters constitute the two *Gene Therapy Protocols* volumes, somewhat artificially separated into Volume 1: *Production and *In Vivo* Applications of Gene Transfer Vectors* and ‘Volume 2: *Design and Characterization of Gene transfer Vectors*. Each chapter includes a brief introduction and practical sections designed to be directly usable at the bench as much as possible. A valuable feature is the note section in which comments and troubleshooting tips are given, allowing new cooks to benefit from the experience of renowned chefs. This pattern is perfect for most chapters describing methods, but may be less fit for chapters in which applications are described. Those may have been clearer and lighter with a simple report format and cross-reference with the methodological chapters.

Non-viral vectors are qualitatively well represented in the two volumes, although they represent a minority of chapters. The contributions on these technologies are of the highest level, with detailed and thorough protocols. Most chapters (27/40) describe viral vector-based technologies: gammaretroviruses and lentiviruses, adenoviruses (including the more advanced ‘helper-dependent’ kind), parvoviruses (adeno-associated viruses (AAVs)) and a single chapter for herpesviruses that reflect the marginal use of this vector family. Among these, authoritative contributions by leaders in the field can be found, but they are sometimes diluted by chapters with less informational content. Those chapters might have been useful for true beginners and students, but most readers will not need explanations at the level of an inaccurate high school textbook. For instance: *‘…*the virus delivers this DNA cassette to the host cell nucleus and the reporter genes get integrated within the host genome. As the host genome replicates, the messenger RNA (mRNA) for the reporter gene combination also replicates (sic). Then the mRNA molecules are translated to proteins within the cytoplasm*’* (legend to figure 1 in volume 1, page 180).

Another editorial weakness is that most chapters contain their own variation of a vector preparation protocol, with, for instance, eight different protocols for *γ*-retroviral vector preparation. The reader is, therefore, faced with the difficulty of choosing for reference. In addition, beginners in the field may be left with the idea that the success of the application methods is associated with slight variations (if not black magic) in the production method. Instilling this notion may be dangerous and may work against the goal of having standardised, quantifiable and comparable methods in the field.

Inevitably perhaps, the balance of the different vector types is a representation of the field 5 years ago. The latest developments and diversity of lentiviral and AAV vectors do not appear. This is obviously a common problem of this kind of book that tries to bring a broad coverage of a fast evolving technology. In this respect, a well-maintained web version of *Gene Therapy Protocols* would be highly useful to a scientific community who now usually refer to this media when searching for a protocol. An online version could allow access to the illustrations of the book, which are generally of a high quality and which would certainly be appreciated by the readership.

These two volumes give a good overview of the busy field of gene transfer vector research. Putting together in a clear format such a variety of approaches and expertises is not a small feat, and the benefit of the enterprise is not diminished by the unavoidable editorial imperfections. The result is the two volumes that will certainly not stay idle on the library shelf.

